# A Distributed Radio Beacon/IMU/Altimeter Integrated Localization Scheme with Uncertain Initial Beacon Locations for Lunar Pinpoint Landing

**DOI:** 10.3390/s20195643

**Published:** 2020-10-02

**Authors:** Rongjun Mu, Yuntian Li, Rubin Luo, Bingzhi Su, Yongzhi Shan

**Affiliations:** 1 School of Astronautics, Harbin Institute of Technology, Harbin 150001, China; murjun@hit.edu.cn (R.M.); 15B918082@hit.edu.cn (B.S.); zhiyong0451@sina.com (Y.S.); 2 Institute of Aerospace Systems Engineering, Beijing 100076, China; armybin@163.com; 3 Aviation Ammunition Institute, NORINCO Group, Harbin 150001, China

**Keywords:** lunar lander, radio beacons, sparse extended information filter, adaptive filter, integrated navigation

## Abstract

As a growing number of exploration missions have successfully landed on the Moon in recent decades, ground infrastructures, such as radio beacons, have attracted a great deal of attention in the design of navigation systems. None of the available studies regarding integrating beacon measurements for pinpoint landing have considered uncertain initial beacon locations, which are quite common in practice. In this paper, we propose a radio beacon/inertial measurement unit (IMU)/altimeter localization scheme that is sufficiently robust regarding uncertain initial beacon locations. This scheme was designed based on the sparse extended information filter (SEIF) to locate the lander and update the beacon configuration at the same time. Then, an adaptive iterated sparse extended hybrid filter (AISEHF) was devised by modifying the prediction and update stage of SEIF with a hybrid-form propagation and a damping iteration algorithm, respectively. The simulation results indicated that the proposed method effectively reduced the error in the position estimations caused by uncertain beacon locations and made an effective trade-off between the estimation accuracy and the computational efficiency. Thus, this method is a potential candidate for future lunar exploration activities.

## 1. Introduction

Safe and soft pinpoint landing (within 100 m at 3σ from the target site [1]) on an extraterrestrial body has been a central objective since the beginning of human space exploration missions. To date, many manned and unmanned landers have successfully landed on moons (Apollo and Chang’E), planets (Curiosity and Opportunity) and asteroids (Rosetta and Hayabusa-2), with landing footprints in the scale of kilometers [2,3]. Among all these celestial bodies, a growing number of exploration missions will land on the Moon in the future, as it is the most suitable outpost for deep space exploration. A robotic lunar base followed by a human base will likely be constructed during these missions, which will be the first potential implementation of co-located pinpoint landing [4]. With the existence of these infrastructures, the natural consequence is to make use of more ground sources to enhance the current onboard navigation capability for lunar landing missions using, for instance, radio beacons.

Radiometric localization is an extensively used technology on Earth. Although there are many specific kinds of signals and modulations for different applications, the information provided by radio beacons can be divided into three categories [5]: (a) range measurements, (b) range rate measurements and (c) bearing measurements. All of these have drawn attention to improving the accuracy of pinpoint landing. In 2008, NASA proposed a radio measurement-enhanced navigation architecture based on lunar relay satellites (LRS) and lunar communication terminals (LCT) for the Autonomous Landing and Hazard Avoidance Technology (ALHAT) project [6]. By combining range and range rate measurements from LRS and LCT with other basic measurements of the ALHAT sensor suite, the landing accuracy could reach a level of 10 m at 3σ [7].

Several studies regarding the optimization of beacon configurations have been presented, both in terms of the filter accuracy [7,8] and observabilities [9,10]. On this basis, Theil et al. from the German Space Center (Deutsches Zentrum für Luft- und Raumfahrt, DLR) investigated the impact of one to four ground beacons for the small integrated navigation for planetary exploration (SINPLEX) project of ESA [4,11]. In this research, bearing measurements, as well as range and range rate measurements, were utilized by the navigation filter to augment the navigation baseline of SINPLEX. Different cases of beacon locations and setups were compared in the Autonomous Terrain-Based Optical Navigation (ATON) project of DLR, which indicated that the impact of additional bearing measurements was quite small.

All studies listed above were based on a strong assumption that uncertainties only existed in the sensor measurements, which means that all beacons were stationary and their locations were precisely pre-determined. In fact, most ground beacons can only be located either by orbiters or by the deep space network on the order of hundreds of meters [12]. Without the protection of the atmosphere, the diurnal temperature variation on the Moon is more than 300 ${}^{{}^{\circ}}$C. If radio beacons are stationary, meaning that they will be exposed to a harsh environment for a long time, the onboard electronic equipment will be fatally damaged. One possible solution is to maintain radio beacons in the lunar base and redeploy them before each landing mission. Such scenarios serve as a strong motivation to investigate a novel navigation scheme that can locate the lander and update all beacon locations at the same time, which is similar to the range-only simultaneous localization and mapping (RO-SLAM) approach in the field of robotics.

Almost all existing radio beacons/inertial measurement unit (IMU)-integrated navigation algorithms in pinpoint landing are formulated using the state by the covariance matrix Σ and the mean vector μ of the multivariate Gaussian distribution and tracked via the extended Kalman filter (EKF) [4,11,13,14,15] or unscented Kalman filter (UKF) [16,17]. However, the computational complexity and memory space of these algorithms are both quadratic to the dimension of state vectors [18], which makes them unsuitable for dealing with this SLAM-like localization problem.

A large amount of strategies for RO-SLAM [19,20,21,22,23,24] have been developed in recent decades, in which the sparse extended information filter (SEIF) proposed by Thrun et al. [18] has been widely used. Unlike Kalman filters, SEIF represents the state by the information matrix $\Lambda =\Sigma^{-1}$ and the information vector η=Λμ, which makes the update stage more efficient due to the natural sparseness of the information matrix [25]. A conditional sparsification operation is implemented before the prediction stage to maintain the sparse representation of Λ, which leads to a constant execution time online.

While SEIF has been demonstrated to be more efficient and scalable in many applications [26,27,28], the consistency and accuracy of SEIF may be worse than EKF due to three approximations [29]: (a) the linearization of the motion and measurement models, (b) the recovery of the mean vector by iterations and (c) the sparsification via approximated conditional independence. Several methods have been designed to refine the performance of SEIF, such as exactly SEIF (ESEIF) [30], exactly sparse delayed-state filter (ESDSF) [31] and hybrid SEIF (HSEIF) [32]; each method only addresses one aspect of the approximation errors.

Iterated versions of EKF and UKF can increase their consistency and robustness against approximation errors compared to the original versions [33]; however, the rate of convergence is frustrated due to the dense covariance matrix. Due to the sparseness of the information filter, the computational burden is no longer a problem for iterated SEIF. He et al. [34] proposed the first iterated SEIF (ISEIF) for autonomous underwater vehicles, which utilized the Gauss–Newton algorithm for measurement updates rather than the traditional linear combination of predictions and innovations. However, divergence may occur in the Gauss–Newton algorithm when the initial values are far from the optimal values or the coefficient matrix in the normal equation is approximately singular [35].

In addition to the efficiency, the update stage in SEIF is also additive, which makes it capable of deployment in distributed systems. Torres et al. [23] first proposed a distributed RO-SLAM scheme based on SEIF for an autonomous ground vehicle (AGV). The prediction stage and update stage are implemented on the AGV and surrounding beacons, respectively. Except for the traditional direct robot–beacon measurements, additional inter-beacon measurements are also integrated into the SEIF, which reduces uncertainty both in the map estimation and localization accuracy. This work was then extended to a 3D resource-constrained operation with an auxiliary selection tool which only integrates the most informative measurements into the SEIF [36]. Further analysis regarding the effect of inter-beacon measurements on the building of an information matrix followed in [37], validating the preservation of the main properties of SEIF.

Beacon initialization is another challenge in RO-SLAM due to the partial observability of range measurements. As a multi-mode problem, the initial estimation of beacon locations can be obtained either by delayed or undelayed initialization methods. The trilateration method [38,39,40] is the most simple and efficient delayed initialization method; however, it suffers from measurement noise and the odometry error of robots. Olson et al. [41] attempted to address this problem with a 2D probability grid, with a performance that was limited by the size and resolution of the grid.

The shortcomings of these two methods resulted in them being quickly replaced by another widely used delayed initialization method: particle filters (PFs) [23,36,37,42,43]. PFs model the probability density of the beacon location as a uniform distribution around the measurement circle, and then the weight of each particle is updated and finally converges to a single solution by accumulating successive range measurements. Even though PFs can provide more accurate results, their significant computational burden cannot be neglected, which leads to several corresponding investigations [44,45].

In contrast to the delay initialization method, the initial beacon location is modeled with a multi-hypothesis distribution, which makes it available to integrate range measurements from the start. Blanco et al. [46] first proposed a Gaussian mixture model (GMM)-based initialization strategy, which provides accurate results but makes the integration of inter-beacon measurements impossible as each beacon is inserted in an independent EKF. On the basis of this research, Felipe et al. [47] proposed a centralized EKF-based initialization framework, which allowed the integration of inter-beacon measurements, and they enhanced it with a outlier rejection filter [48,49]. Geneve et al. [50] proposed a short-delayed composite initialization method based on Euclidean parameterization and a two-hypothesis GMM, which showed a lower computational cost.

Based on all the existing studies listed above, a novel distributed radio beacon/IMU-integrated localization scheme based on an adaptive iterated sparse extended hybrid filter (AISEHF) is presented in this paper. To the best of our knowledge, this is the first time that beacon location errors have been taken into consideration in the integrated navigation architecture for lunar pinpoint landing. This scheme is inspired by the SEIF-based distributed framework sketched in [37] and the ISEIF proposed in [34], while several aspects have been modified according to the characteristics of lunar pinpoint landing:

- Only lander-beacon range measurements are utilized as inter-beacon measurements and are easily blocked by obstacles, such as craters on the lunar surface.
- A batch-least-squares trilateration algorithm similar to [39] was adopted for the initialization of beacon locations, as their prior rough estimations are available and the inertial measurement unit (IMU) used in our application is much more accurate than that used in the majority of RO-SLAM applications.
- The navigation state between two successive measurements was propagated by a hybrid form of the “mean vector + information matrix” to avoid the frequent conversion from the information vector to the mean vector, which can improve the computational efficiency of the prediction stage.
- An adaptive iteration algorithm with the damping factor was adopted in the update stage, which can both improve the accuracy and efficiency of the estimator.

The remainder of this paper is structured as follows. The problem formulation is given in Section 2. In Section 3, we detail the implementations of the proposed distributed localization scheme. In Section 4, we verify the performance of the proposed algorithm by simulations, which is followed by further discussions. Eventually, our conclusions are presented in Section 5.

## 2. Problem Formulation

**Figure 1:**
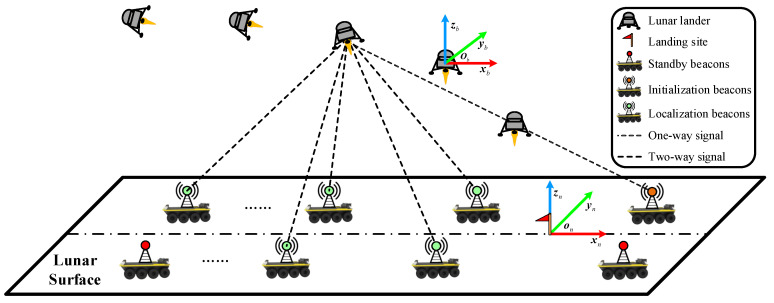
Overview of the proposed distributed radio beacon/inertial measurement unit (IMU)-integrated localization scheme and definitions of the landing frame L (east, north and up (ENU)) and the body frame B (front–left–up (FLU)).

As illustrated in Figure 1, the target application scenario of this paper is the final phase of the power descent, which is short both in terms of the flight time and downrange distance (commonly around 200 s and 5–10 km). Without generality, the landing frame (L) can be chosen as the navigation frame. The origin of the L frame $o_{l}$ is located at the targeted landing site, and three coordinate axes ($x_{l}$, $y_{l}$, and $z_{l}$) are aligned with the geographic directions east, north and up (ENU), respectively. Another significant coordinate system is the body frame B, whose origin lies in the mass center of the lander, and the directions of the three axes are front–left–up (FLU); i.e., the $x_{b}$ axis points to the front along the body symmetric axis, the $y_{b}$ axis points to the left and is orthogonal to $x_{b}$ and the $z_{b}$ axis completes the right-handed orthogonal coordinate system. In addition, measurements of the laser altimeter are present in the sensor frame (S), whose origin and $x_{s}$ axis are the same as the B frame but whose $y_{s}$ axis and $z_{s}$ axis are opposite to $y_{b}$ and $z_{b}$.

The kinematic equations of the lunar lander, defined with respect to the landing frame L, are given as follows: [51]:

(1) $$\left\{\begin{array}{cc} {\dot{p}}^{L} & =v^{L}\\ {\dot{v}}^{L} & =C_{B}^{L}f_{i}^{B}-{\left[2\omega_{im}^{L}\right]}^{\wedge }v^{L}+g^{L}\\ {\dot{C}}_{B}^{L} & =C_{B}^{L}{\left[\omega_{ib}^{B}\right]}^{\wedge } \end{array}\right.$$

where ${\left[\cdot \right]}^{\wedge }$ is the skew symmetric operator; $p^{L}$ is the lander position; $v^{L}$ is the lander velocity; $C_{B}^{L}$ is the rotation matrix from B to L; $f_{i}^{B}$ and $\omega_{ib}^{B}$, respectively, denote the specific force and the angular rate sensed by IMU, whose further interpretations can be found in [52]; and $g^{L}$ is the gravity vector, whose magnitude is assumed to vary with the altitude as follows [51]:

(2) $$∥g∥=\frac{g_{0}}{{\left(1+h/R_{m}\right)}^{2}}$$

where $g_{0}=1.622$ m/s${}^{2}$ is the gravity constant at the lunar surface, *h* is the current altitude of the lander and $R_{m}$ is the radius of the Moon. The superscripts that denote the corresponding frame are omitted for notational convenience in the following parts if there is no confusion.

The beacon receiver is attached on the bottom of the lander, which makes it directly visible from the ground beacons. Several radio beacons are mounted on rovers and pre-deployed along the designed ground track of the landing mission, while their prior rough locations are determined by the onboard navigation systems of the rover. Due to the limited effect of bearing measurements, each beacon is assumed to be equipped with a range-only sensor whose measurements $r_{li}$ are affected by independent Gaussian white noise $v_{b}$:

(3) $$\left\{\begin{array}{cc} r_{li} & ={\bar{r}}_{li}+v_{b},\;v_{b}∼N\left(0,\sigma_{b}\right)\\ {\bar{r}}_{li} & =\sqrt{\delta x^{2}+\delta y^{2}+\delta z^{2}}=\sqrt{{\left(x_{l}-x_{b_{i}}\right)}^{2}+{\left(y_{l}-y_{b_{i}}\right)}^{2}+{\left(z_{l}-z_{b_{i}}\right)}^{2}} \end{array}\right.$$

where $p_{l}=\left[x_{l},y_{l},z_{l}\right]$ and $p_{b_{i}}=\left[x_{b_{i}},y_{b_{i}},z_{b_{i}}\right]$, respectively, denote the positions of the lander and the *i*th beacon. In addition, only the lander-beacon range measurements are available, as there are too many obstacles (such as craters or huge rocks) on the lunar surface, which could heavily degrade the inter-beacon measurements. Considering the terrain accessibility of the rover, all ground beacons are assumed to be on the same horizontal plane. This coplanarity will lead to uncertainty in altitude estimation; thus, a laser altimeter is mounted next to the beacon receiver to offer additional range measurements $r_{a}$:

(4) $$r_{a}=\frac{z_{l}}{\cos\gamma \cos\theta }+v_{a},\;v_{a}∼N\left(0,\sigma_{a}\right)$$

where θ is the pitch angle, γ is the roll angle and $v_{a}$ is the Gaussian white noise.

**Figure 2:**
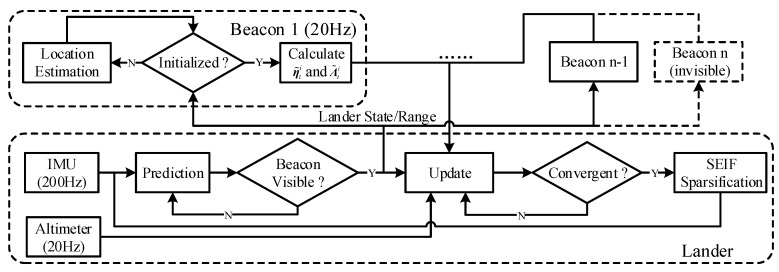
The flow diagram of the tightly-coupled navigation system based on the adaptive iterated sparse extended hybrid filter (AISEHF).

The flow diagram of the proposed distributed localization scheme is shown in Figure 2, which can be divided into two parts: the lander and the beacon. The prediction stage is continuously conducted onboard based on the IMU measurements. After the visible beacons have been detected, a message that contains the current estimated lander position ${\hat{p}}_{l}$ will be transmitted to all visible beacons. When this message has been received by beacon $b_{i}$, it will switch from the “Standby” mode to the “Initialization” mode, in which the beacon location is initialized by minimizing the following batch-least-squares cost function with *N* range measurements:

(5) $$p_{b_{i}}^{*}=\arg\min_{p_{b_{i}}}\sum_{k=1}^{N}{∥r_{li}^{k}-\sqrt{{\left({\hat{p}}_{l}^{k}-p_{b_{i}}\right)}^{T}\left({\hat{p}}_{l}^{k}-p_{b_{i}}\right)}∥}^{2}$$

where *N* can be pre-determined offline and Equation (5) can be solved by the nonlinear optimization methods given in [53] with prior rough beacon locations as initial values.

When the initialization of $b_{i}$ is convergent, it starts to work in the “Localization” mode. Its contribution (local information vector ${\tilde{\eta }}_{t}^{i}$ and information matrix ${\tilde{\Lambda }}_{t}^{i}$) to the update stage is calculated and sent back to the lander. Then, the locations of all visible beacons are inserted into the state vector, and their contributions are summed together as follows:

(6) $${\tilde{\eta }}_{t}={\hat{\eta }}_{t}+\sum_{i=1}^{k}S_{i}{\tilde{\eta }}_{t}^{i},\;{\tilde{\Lambda }}_{t}={\hat{\Lambda }}_{t}+\sum_{i=1}^{k}S_{i}{\tilde{\Lambda }}_{t}^{i}S_{i}^{T}$$

where $\hat{\left(\cdot \right)}$ and $\tilde{\left(\cdot \right)}$ denote the variables obtained by the state prediction and measurement update, respectively. $S_{i}={\left[\begin{array}{ccccc} 0 & \cdots & I & \cdots & 0 \end{array}\right]}^{T}$ is a projection matrix that allocates ${\tilde{\eta }}_{t}^{i}$ and ${\tilde{\Lambda }}_{t}^{i}$ at the corresponding position of ${\tilde{\eta }}_{t}$ and ${\tilde{\Lambda }}_{t}$:

(7) $$S_{i}{\tilde{\eta }}_{t}^{i}=\left[\begin{array}{c} 0\\ \vdots \\ {\tilde{\eta }}_{t}^{i}\\ \vdots \\ 0 \end{array}\right],\;S_{i}{\tilde{\Lambda }}_{t}^{i}S_{i}^{T}=\left[\begin{array}{ccccc} 0 & \cdots & 0 & \cdots & 0\\ \vdots & \ddots & \vdots & \; & \vdots \\ 0 & \cdots & {\tilde{\Lambda }}_{t}^{i} & \cdots & 0\\ \vdots & \; & \vdots & \ddots & \vdots \\ 0 & \cdots & 0 & \cdots & 0 \end{array}\right].$$

Finally, an iterated update was performed based on the global information vector ${\tilde{\eta }}_{t}$ and information matrix ${\tilde{\Lambda }}_{t}$, which was followed by the sparsification step of SEIF when the number of visible beacons were out of bounds or some links in the information matrix were too weak [34].

## 3. Adaptive Iterated Sparse Extended Hybrid Filter

### 3.1. State Definition and Jacobians

Like EKF, the first-order Taylor series expansion was applied to linearize the state space model as follows in Equation (8) to deal with nonlinear propagation and measurement functions, where $w_{t}$ and $v_{t+1}$ are the white process noise and measurement noise, respectively.

(8) $$\left\{\begin{array}{cc} \xi_{t+1} & =f\left(\xi_{t},u_{t}\right)+w_{t}\approx F_{t}\xi_{t}+G_{t}u_{t}+w_{t},\;w_{t}∼N\left(0,Q_{t}\right)\\ z_{t+1} & =h\left(\xi_{t+1}\right)+v_{t+1}\approx H_{t+1}\xi_{t+1}+v_{t+1},\;v_{t+1}∼N\left(0,R_{t+1}\right) \end{array}\right.$$

where $\xi_{t}={\left[x_{t}^{T},B^{T}\right]}^{T}$ denotes the state vector at time *t* comprised of the lander state $x_{t}={\left[p_{lt}^{T},v_{lt}^{T}\right]}^{T}$ and the set of visible beacons locations $B={\left[p_{b_{1}}^{T},\cdots ,p_{b_{n}}^{T}\right]}^{T}$. As a navigation (or localization) filter, it is common to choose the specific force $f_{i}$ and gravity vector g as the control input $u_{t}={\left[f_{i}^{T},g^{T}\right]}^{T}$.

As B is kept unchanged and is independent of $x_{t}$, we compute the Jacobians F and G from the kinematic model (Equation (1) with respect to the $x_{t}$, which is more efficient. The results are given as follows:

(9) $$F=\frac{\partial f(\xi ,u)}{\partial x}=\left[\begin{array}{cc} I & \Delta tI\\ 0 & I-\Delta t{\left[2\omega_{im}^{L}\right]}^{\wedge } \end{array}\right]$$

(10) $$G=\frac{\partial f(\xi ,u)}{\partial u}=\left[\begin{array}{cc} 0 & 0\\ \Delta tC_{B}^{L} & \Delta tI \end{array}\right].$$

Similarly, based on the measurement function of Equation (3) and (4), the measurement Jacobian H is given in Equations (11)∼(13), where all entries that are not given are zero.

(11) $$H=\left[\begin{array}{c} H_{li}\\ \vdots \\ H_{lj}\\ H_{a} \end{array}\right]=\left[\begin{array}{ccccccc} \frac{\partial r_{li}}{\partial x_{t}} & \cdots & \frac{\partial r_{li}}{\partial p_{b_{i}}} & \cdots & 0 & \cdots & 0\\ \vdots & \; & \vdots & \ddots & \vdots & \; & \vdots \\ \frac{\partial r_{lj}}{\partial x_{t}} & \cdots & 0 & \cdots & \frac{\partial r_{lj}}{\partial p_{b_{j}}} & \cdots & 0\\ \frac{\partial r_{a}}{\partial x_{t}} & \cdots & 0 & \cdots & 0 & \cdots & 0 \end{array}\right]$$

(12) $$H_{li}=\left[\begin{array}{cccccccc} \frac{\delta x}{r_{li}} & \frac{\delta y}{r_{li}} & \frac{\delta z}{r_{li}} & \cdots & -\frac{\delta x}{r_{li}} & -\frac{\delta y}{r_{li}} & -\frac{\delta z}{r_{li}} & \cdots \end{array}\right]$$

(13) $$H_{a}=\left[\begin{array}{cccc} 0 & 0 & \frac{1}{\cos\gamma \cos\theta }\; & \cdots \end{array}\right].$$

### 3.2. Temporal Alignment of Asynchronous Measurements

**Figure 3:**
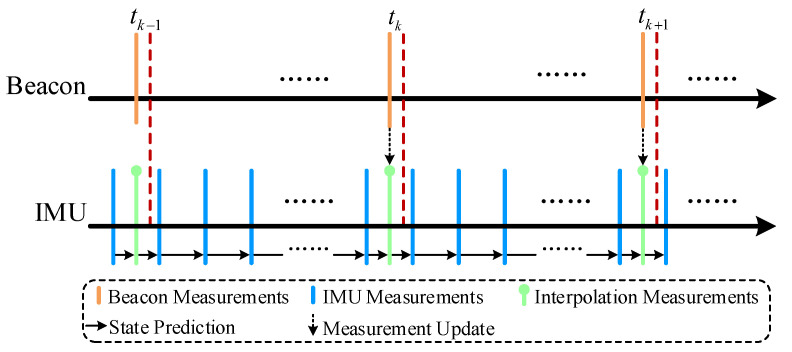
An example of processing asynchronous inertial measurement unit (IMU) and beacon measurements.

Signal transmission or triggering delays will lead to a temporal misalignment between measurements from different sensors, which cannot be ignored in the integrated localization scheme. As depicted in Figure 3, a virtual measurement method based on linear interpolations was adopted in our scheme to eliminated the temporal misalignment. Assuming there are *j* IMU measurements between every two sequential beacon measurements and the two closest IMU measurements to $t_{k}$ are denoted as $m_{t_{k-1/j}}^{i}$ and $m_{t_{k/1}}^{i}$, the virtual measurement at $t_{k}$ can be obtained as follows:

(14) $$m_{t_{k}}^{i}=\frac{t_{k/1}-t_{k}}{t_{k/1}-t_{k-1/j}}m_{t_{k-1/j}}^{i}+\frac{t_{k}-t_{k-1/j}}{t_{k/1}-t_{k-1/j}}m_{t_{k/1}}^{i}.$$

When all measurements are temporally aligned, the state prediction and measurement update stage of SEIF are then executed separately.

### 3.3. Hybrid State Prediction

The typical prediction stage in SEIF can be separated into two main processes [31]—state augmentation and state marginalization—assuming the state vector $\xi_{t}$ is subjected to the conditional distribution given, $z_{t}$ and $u_{t-1}$, as expressed in Equation (15):

(15) $$p\left(\xi_{t}|z_{t},u_{t-1}\right)∼N\left(\left[\begin{array}{c} \mu_{x_{t}}\\ \mu_{B} \end{array}\right],\left[\begin{array}{cc} \Sigma_{x_{t}x_{t}} & \Sigma_{x_{t}B}\\ \Sigma_{Bx_{t}} & \Sigma_{BB} \end{array}\right]\right)∼N^{-1}\left(\left[\begin{array}{c} \eta_{x_{t}}\\ \eta_{B} \end{array}\right],\left[\begin{array}{cc} \Lambda_{x_{t}x_{t}} & \Lambda_{x_{t}B}\\ \Lambda_{Bx_{t}} & \Lambda_{BB} \end{array}\right]\right).$$

At time t+1, a new variable $x_{t+1}$, which represents the newest lander state, is first inserted into the state vector $\xi_{t}$, and the augmented distribution $p\left(x_{t},x_{t+1},B\right)∼N^{-1}\left({\bar{\eta }}_{t+1},{\bar{\Lambda }}_{t+1}\right)$ is given as follows: 1.5

(16) $${\bar{\Lambda }}_{t+1}=\left[\begin{array}{ccc} \Lambda_{x_{t}x_{t}}+F^{T}Q_{t}^{-1}F & -F^{T}Q_{t}^{-1} & \Lambda_{x_{t}B}\\ -Q_{t}^{-1}F & Q_{t}^{-1} & 0\\ \Lambda_{Bx_{t}} & 0 & \Lambda_{BB} \end{array}\right]=\left[\begin{array}{cc} {\bar{\Lambda }}_{t+1}^{\alpha \alpha } & {\bar{\Lambda }}_{t+1}^{\alpha \beta }\\ {\bar{\Lambda }}_{t+1}^{\beta \alpha } & {\bar{\Lambda }}_{t+1}^{\beta \beta } \end{array}\right]$$

(17) $${\bar{\eta }}_{t+1}=\left[\begin{array}{c} \eta_{x_{t}}-F^{T}Q_{t}^{-1}\left(f\left(\mu_{x_{t}},u_{t}\right)-F\mu_{x_{t}}-Gu_{t}\right)\\ Q_{t}^{-1}\left(f\left(\mu_{x_{t}},u_{t}\right)-F\mu_{x_{t}}-Gu_{t}\right)\\ \eta_{B} \end{array}\right]=\left[\begin{array}{c} {\bar{\eta }}_{t+1}^{\alpha }\\ {\bar{\eta }}_{t+1}^{\beta } \end{array}\right].$$

The propagated distribution $p\left(x_{t+1},B|z_{t},u_{t}\right)$ can then be recovered by marginalizing out $x_{t}$ from the joint distribution $p\left(x_{t},x_{t+1},B|z_{t},u_{t}\right)$:

(18) $${\hat{\Lambda }}_{t+1}={\bar{\Lambda }}_{t+1}^{\beta \beta }-{\bar{\Lambda }}_{t+1}^{\beta \alpha }{\left({\bar{\Lambda }}_{t+1}^{\alpha \alpha }\right)}^{-1}{\bar{\Lambda }}_{t+1}^{\alpha \beta }$$

(19) $${\hat{\eta }}_{t+1}={\bar{\eta }}_{t+1}^{\beta }-{\bar{\Lambda }}_{t+1}^{\beta \alpha }{\left({\bar{\Lambda }}_{t+1}^{\alpha \alpha }\right)}^{-1}{\bar{\eta }}_{t+1}^{\alpha }.$$

From Equations (16) and (17), the recovery of $\mu_{t}$ from $\eta_{t}$ must be performed before the state augmentation to re-linearize F and G [18]. This is not a problem for a synchronous application, as $\eta_{t}$ will be utilized in the following update stage, whereas in an asynchronous application, the prediction stage will be followed by several other prediction stages, meaning that the state recovery will be implemented several times. As the state recovery is commonly performed based on iterated algorithms, as with a pre-conditioned gradient, these extra steps will lead to an unnecessary computational burden.

Based on the analysis above, we proposed a hybrid form of the “mean vector + information matrix” for the prediction stage, which will be called the sparse extended hybrid filter (SEHF) in the following. SEHF propagates the uncertainty of $\xi_{t}$ using $\Lambda_{t}$ as with the typical SEIF, while the expectation of $\xi_{t}$ is propagated by $\mu_{t}$ instead of $\eta_{t}$ as follows:

(20) $${\hat{\mu }}_{t+1}=\left[\begin{array}{cc} F & 0\\ 0 & I \end{array}\right]\mu_{t}+\left[\begin{array}{c} G\\ 0 \end{array}\right]u_{t}=\left[\begin{array}{c} F\mu_{x_{t}}+Gu_{t}\\ \mu_{B} \end{array}\right].$$

**Figure 4:**
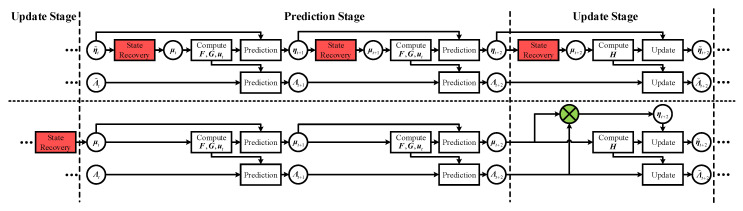
Comparison of the typical form and our hybrid form prediction. Different processing steps between these two forms are marked with different colors depending on their computational burden.

Figure 4 shows a comparison of the typical form and our hybrid form prediction when there are two prediction stages between successive update stages. Clearly, in the hybrid form, the state recovery only needs to be performed once before the filter switches from the update stage to the prediction stage, regardless of the number of extra prediction stages, whereas it will increase with the number of extra prediction stages in the typical form. Although there is an extra operation in the hybrid form that constructs $\eta_{t}$ for the update stage, the corresponding computational cost is quite small, as this is a simple matrix multiplication.

### 3.4. Adaptive Iterated Measurement Update

#### 3.4.1. Iterated Measurement Update

When the current measurement $z_{t+1}$ is achieved, the SEIF typically updates the new posterior $p\left(\xi_{t+1}|z_{t+1},u_{t}\right)$ according to the chain rule based on existing distributions $p\left(\xi_{t+1}|z_{t},u_{t}\right)∼N^{-1}\left({\hat{\eta }}_{t+1},{\hat{\Lambda }}_{t+1}\right)$ and $p\left(z_{t+1}|\xi_{t+1}\right)∼N\left(h\left(\xi_{t+1}\right),R_{t+1}\right)$:

(21) $${\tilde{\Lambda }}_{t+1}={\hat{\Lambda }}_{t+1}+H^{T}R_{t+1}^{-1}H$$

(22) $${\tilde{\eta }}_{t+1}={\hat{\eta }}_{t+1}+H^{T}R_{t+1}^{-1}\left(z_{t+1}-h\left({\hat{\mu }}_{t+1}\right)+H{\hat{\mu }}_{t+1}\right).$$

Varying from solving the measurement update via a linear combination of the prediction and innovation in the traditional filter, the iterated filter models it as a nonlinear least squares (NLS) problem as follows:

(23) $$\begin{array}{cc} \xi_{t+1}^{*}= & \arg\min_{\xi_{t+1}}\left\{\frac{1}{2}{\left(z_{t+1}-h\left(\xi_{t+1}\right)\right)}^{T}R_{t+1}^{-1}\left(z_{t+1}-h\left(\xi_{t+1}\right)\right)\right.\\ + & \left.\frac{1}{2}{\left(\xi_{t+1}-{\hat{\mu }}_{t+1})\right)}^{T}{\hat{\Sigma }}_{t+1}^{-1}\left(\xi_{t+1}-{\hat{\mu }}_{t+1})\right)\right\}\\ = & \arg\min_{\xi_{t+1}}\chi^{2}\left(\xi_{t+1}\right) \end{array}$$

where $\chi^{2}\left(\xi_{t+1}\right)$ is the objective function of the NLS problem and $\xi_{t+1}^{*}$ is the optimal value when $\chi^{2}\left(\xi_{t+1}\right)$ takes the minimum value, defined by the following matrices:

(24) $$Z=\left[\begin{array}{c} z_{t+1}\\ {\hat{\mu }}_{t+1} \end{array}\right],\;G\left(\xi_{t+1}\right)=\left[\begin{array}{c} h\left(\xi_{t+1}\right)\\ \xi_{t+1} \end{array}\right],\;W=\left[\begin{array}{cc} R_{t+1}^{-1} & 0\\ 0 & {\hat{\Sigma }}_{t+1}^{-1} \end{array}\right].$$

The objective function $\chi^{2}\left(\xi_{t+1}\right)$ can be rewritten into the following a matrix form [33]:

(25) $$\begin{array}{cc} \chi^{2}\left(\xi_{t+1}\right)\; & =\frac{1}{2}{\left(Z-G(\xi_{t+1})\right)}^{T}W\left(Z-G(\xi_{t+1})\right)\\ \; & =\frac{1}{2}\underset{e^{T}\left(\xi_{t+1}\right)}{\underset{︸}{{\left(Z-G(\xi_{t+1})\right)}^{T}C^{T}}}\underset{e(\xi_{t+1})}{\underset{︸}{C\left(Z-G(\xi_{t+1})\right)}}\\ \; & =\frac{1}{2}{∥e\left(\xi_{t+1}\right)∥}^{2} \end{array}.$$

Then, the iterative sequences of $\xi_{t+1}^{i}$ and $\Lambda_{t+1}^{i}$ can be achieved as follows [34]:

(26) $$K^{i}={\left(H_{i}^{T}R^{-1}H_{i}+{\hat{\Lambda }}_{t+1}\right)}^{-1}H_{i}^{T}R^{-1}$$

(27) $$\xi_{t+1}^{i+1}={\hat{\mu }}_{t+1}+K^{i}\left[z_{k+1}-h\left(\xi_{t+1}^{i}\right)-H_{i}\left({\hat{\mu }}_{t+1}-\xi_{t+1}^{i}\right)\right]$$

(28) $$\Lambda_{t+1}^{i}=H_{i}^{T}R^{-1}H_{i}+{\hat{\Lambda }}_{t+1}$$

where $H_{i}$ is the Jacobian of the measurement function at $\xi_{t+1}^{i}$.

#### 3.4.2. Iterated Measurement Update with Damping Factor

The iterative form update equations given above are obtained by solving the following normal equation of the Gauss–Newton algorithm:

(29) $$J_{i}^{T}J_{i}\Delta \xi_{t+1}^{i}=-J_{i}^{T}e\left(\xi_{t+1}^{i}\right)=\Delta r_{i}$$

where $J_{i}$ represents the Jacobian of $e(\xi_{t+1})$ with respect to $\xi_{t+1}$ evaluated at $\xi_{t+1}^{i}$. As the coefficient matrix $J_{i}^{T}J_{i}$ is only positive semidefinite, there is no guarantee that Δξ is always along the descent direction. Δξ would become unstable when the initial value was far away from the solution as the second derivative (Hessian) of the object function was approximated by the Jacobian. To address these issues, a damped Gauss–Newton algorithm was proposed by Levenberg and Marquardt, successivel [53]. In this method, a positive damping factor λ was added to the coefficient matrix of the normal equation, which is known as the damping factor:

(30) $$\left(J_{i}^{T}J_{i}+\lambda I\right)\Delta \xi_{t+1}^{i}=\Delta r_{i},\;\lambda >0.$$

Clearly, $\left(J_{i}^{T}J_{i}+\lambda I\right)$ is always positive and the method is equivalent to the steepest descent algorithm and the Gauss–Newton algorithm when $\mu I\gg J_{i}^{T}J_{i}$ and $\mu I\ll J_{i}^{T}J_{i}$, respectively. With this modification, the iterative sequence of $\xi_{t}$ is modified as follows (see Appendix A for more details):

(31) $$K^{i}={\left(H_{i}^{T}R^{-1}H_{i}+{\hat{\Lambda }}_{t+1}+\lambda I\right)}^{-1}H_{i}^{T}R^{-1}$$

(32) $$\left\{\begin{array}{cc} \xi_{t+1}^{i+1} & =\Gamma_{t+1}{\hat{\mu }}_{t+1}+K^{i}\left[z_{t+1}-h\left(\xi_{t+1}^{i}\right)-H\left(\Gamma_{t+1}{\hat{\mu }}_{t+1}-\xi_{t+1}^{i}\right)\right]\\ \Gamma_{t+1} & =\frac{{\hat{\Lambda }}_{t+1}}{\lambda }\left[I-{\left(I+\lambda {\hat{\Lambda }}_{t+1}\right)}^{-1}\frac{{\hat{\Lambda }}_{t+1}}{\lambda }\right] \end{array}\right.$$

As the uncertainty of $\xi_{t}$ will only be updated when the iteration converges [33], the update of $\Lambda_{t}$ is the same as Equation (28). As a hybrid form is adopted for the following prediction stages in our scheme, there is no need to convert $\mu_{t}$ to $\eta_{t}$, which further reduces the computational burden.

#### 3.4.3. Damping Factor and Stopping Criteria

A proper value of λ is vital to the iteration. One possible solution is to choose the initial value of λ depending on the maximum value of the coefficient matrix:

(33) $$\lambda_{0}=\tau \cdot \max\left(diag\left(J_{0}^{T}J_{0}\right)\right),\;\tau \in \left[10^{-8},1\right]$$

and then update λ based on the gain ratio ρ at each iteration [54]:

(34) $$\lambda =\left\{\begin{array}{c} \lambda *\max\left(\frac{1}{3},1-{\left(2\rho -1\right)}^{3}\right),\nu =2;\;\rho >0\\ \lambda *\nu ,\nu =2*\nu ;\;\rho \leq 0 \end{array}\right.$$

where ρ is the ratio of the actual and approximated decrease of the objective function:

(35) $$\rho =\frac{\chi^{2}\left(\xi_{t+1}^{i}\right)-\chi^{2}\left(\xi_{t+1}^{i}+\Delta \xi_{t+1}^{i}\right))}{L\left(0\right)-L\left(\Delta \xi_{t+1}^{i}\right)}$$

(36) $$\begin{array}{cc} L\left(0\right)-L\left(\Delta \xi_{t+1}^{i}\right) & =-{\left(\Delta \xi_{t+1}^{i}\right)}^{T}J_{i}^{T}e\left(\xi_{t+1}^{i}\right)-\frac{1}{2}{\left(\Delta \xi_{t+1}^{i}\right)}^{T}J_{i}^{T}J_{i}\Delta \xi_{t+1}^{i}\\ \; & =\frac{1}{2}{\left(\Delta \xi_{t+1}^{i}\right)}^{T}\left(\Delta r_{i}+\lambda \Delta \xi_{t+1}^{i}\right). \end{array}$$

Another parameter of concern is the number of iterations, which balances the accuracy and efficiency of the iteration process. Commonly, this parameter is difficult to predetermine offline; thus, several adaptive stopping criteria are applied in the proposed algorithm. First, the iteration process should be terminated when the difference between two successive residuals is smaller than a threshold $\epsilon_{1}$, which reflects the global minimum:

(37) $$∥\Delta r_{i+1}-\Delta r_{i}∥<\epsilon_{1}.$$

Another stopping criterion is related to the local minimum; i.e., the increment $\Delta \xi_{t+1}^{i}$ is smaller than the threshold $\epsilon_{2}$:

(38) $$∥\Delta \xi_{t+1}^{i}∥<\epsilon_{2}.$$

Lastly, the maximum iterations $k_{max}$ should be settled in the case of a dead loop.

**Figure 5:**
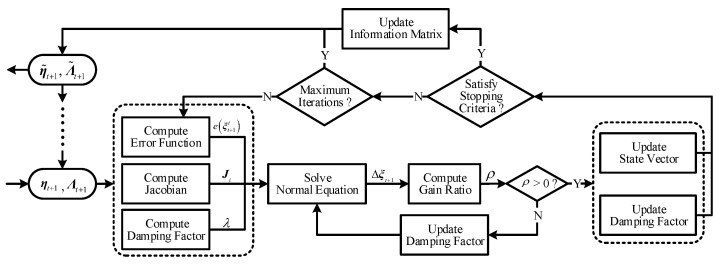
A schematic diagram of the adaptive iterative update with the damping factor.

With all notations and definitions above, the complete working flow of the adaptive iterative update is summarized in Figure 5.

## 4. Results and Discussions

### 4.1. Simulation Scenario and Parameters

**Figure 6:**
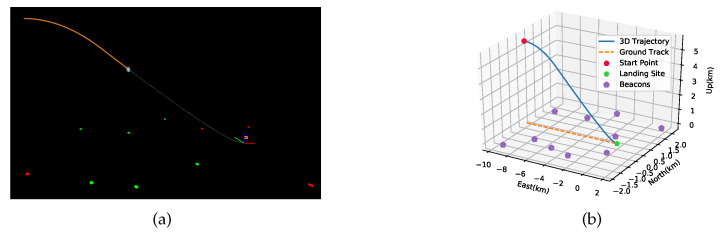
Illustration of the simulation scenario with 10 radio beacons. (**a**) The simulation scenario visualized in OpenGL, where the red flag with yellow edges denotes the desired landing site and points in red and green, respectively, denote standby and working beacons. (**b**) The designed landing trajectory in the L frame.

**Figure 7:**
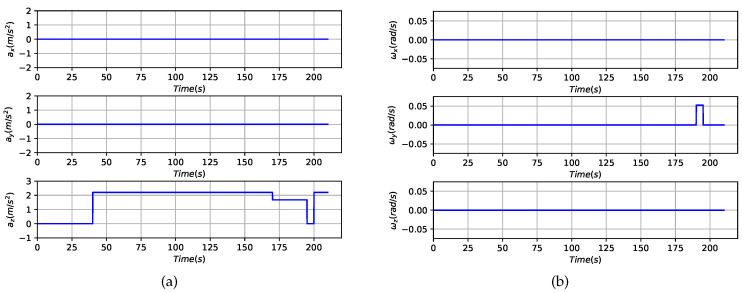
Control profiles of the designed trajectory in the simulation scenarios: (**a**) Triaxial specific force profile in the B frame. (**b**) Triaxial angular rate profile in the B frame.

The simulation scenario is a final landing phase performed after the main break phase of the powered descent, as shown in Figure 6. The landing trajectory was simulated for 210 s with an initial position of $\left[-9797,0,5530\right]$ m, an initial velocity of $\left[85,0,0\right]$ m/s and an initial attitude of $\left[0,-15,0\right]$ in degrees. Figure 7 presents the profile of the specific force and angular rate in detail. Ten beacons were deployed uniformly along both sides of the ground track in the simulation, and their true locations are listed in Table 1. An initial position error of 200 m was added to their true positions to simulate the prior rough locations obtained by rovers.

With respect to the navigation filter, the number of measurements *K* in the beacon initialization in Equation (5) was chosen to be 50. The attitude was propagated based on raw gyroscope measurements and periodically calibrated by the star tracker independently with a residual around 9.1″ [4]. The residuals of the initial position and velocity were set to 300 m and 30 m/s, respectively. The 1σ parameters of IMU, the laser altimeters and ground beacons are listed in Table 2, and their update frequencies are, respectively, settled to 200 Hz, 100 Hz, and 20 Hz.

Other parameters of the localization filter—i.e., the initial covariance matrix $p_{0}$, the process noise covariance matrices $Q_{t}$ and the measurement noise covariance matrices $R_{t+1}$—are given as follows:

(39) $$p_{0}=diag{\left[1\times 10^{4},1\times 10^{4},1\times 10^{4},1\times 10^{2},1\times 10^{2},1\times 10^{2},1\times 10^{4},\cdots ,1\times 10^{4}\right]}_{\left(n+6)\times (n+6\right)}$$

(40) $$Q_{k}=diag{\left[0.5,0.1,5,0.005,0.0001,0.001\right]}_{6\times 6}$$

(41) $$R_{k+1}=diag{\left[1\times 10^{4},\cdots ,1\times 10^{4},25\right]}_{\left(n+1)\times (n+1\right)}$$

where *n* is the number of available beacons.

The simulation system was implemented in C++ using the Robot Operating System (ROS) on a laptop with an Intel Core™i7-4790 @ 3.68 GHz and 8 GB RAM. Each sensor runs an independent ROS node to simulate the real interactions.

### 4.2. Simulation Results and Analyses

**Figure 8:**
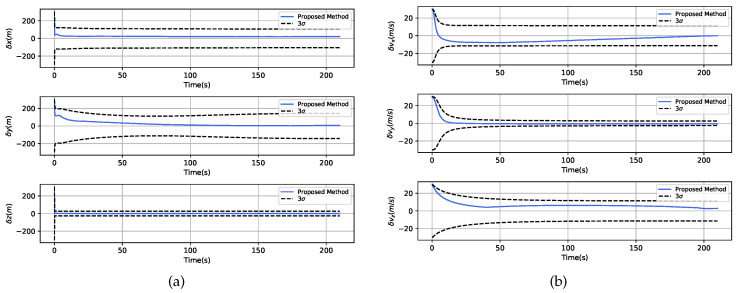
Estimation errors from the proposed tightly-coupled navigation scheme. (**a**) Triaxial position errors with 3σ uncertainty bound. (**b**) Triaxial velocity errors with the 3σ uncertainty bound.

The estimation errors of the navigation state obtained by the proposed AISEHF-based distributed localization scheme are shown in Figure 8. It can be seen from the results that the residuals converged into the 1σ uncertainty bound rapidly both in the triaxial position and velocity estimations with steady values less than 50 m and 5 m/s, which meet the demands of pinpoint landing.

**Figure 9:**
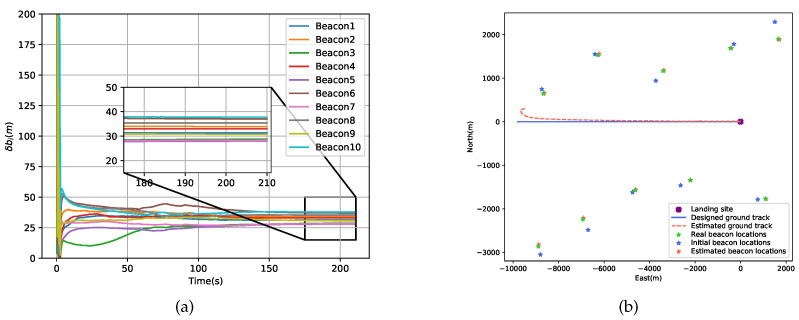
Performance of the proposed scheme in the mapping of the beacon configuration. (**a**) Residuals of all estimated beacon locations. (**b**) The beacon map and ground track before and after the estimation.

Figure 9a presents the performance of the proposed method in the mapping of all ground beacons. Due to the initialization procedure, almost all beacon location errors converged within 50 s, and the average error was 32.41 m. From Figure 9b, there appears to be an offset between the estimated value and the true value both in the beacon locations and the ground track. This appearance is induced by the fact that there is neither an accurate initial position of the lander nor for ground beacons, which means that their absolute locations are not observable.

Then, the proposed scheme (*Scheme 3*) was further compared with another two schemes with the same sensor configurations: (a) the SEIF-based scheme proposed in [37] without inter-beacon measurements (*Scheme 1*) and (b) the ISEIF scheme proposed in [34] (*Scheme 2*). The performance of all three schemes was evaluated in 100 independent Monte Carlo runs. In each of the 100 runs, all random seeds (e.g., beacon locations and sensor noise) were changed randomly, while all parameters of estimator were kept unchanged. Two indicators—root-mean-square errors (RMSEs) and averaged RMSEs (ARMSEs), which are defined in Equation (42)—were adopted to acquire a fair result.

(42) $$\begin{array}{cc} \epsilon_{RMSE}\left(t\right) & =\sqrt{\frac{1}{K}\sum_{i=1}^{K}\frac{{\left({\tilde{x}}_{t}^{i}-x_{t}^{i}\right)}^{T}\left({\tilde{x}}_{t}^{i}-x_{t}^{i}\right)}{n}},\;x\in R^{n}\\ \epsilon_{ARMSE} & =\frac{1}{T}\sum_{t=0}^{T}\epsilon_{RMSE}\left(t\right) \end{array}$$

where *K* is the number of Monte Carlo runs and *T* is the total time from the convergence to the end. ${\tilde{x}}_{t}^{i}$ and $x_{t}^{i}$, respectively, denote the estimated and true state at time *t* in the ith run.

**Figure 10:**
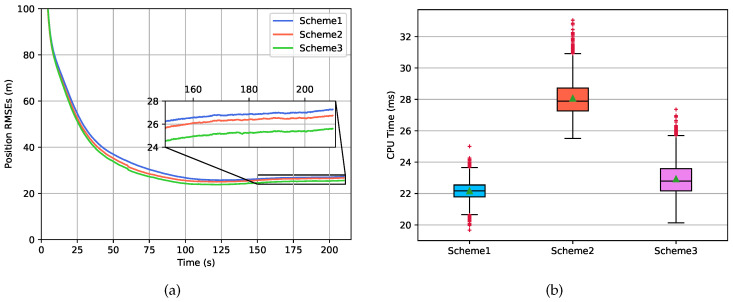
Performance analyses of different localization schemes from 100 independent Monte Carlo runs. (**a**) Root-mean-square errors (RMSEs) in the position estimation. (**b**) Averaged computational cost.

Figure 10 gives the position RMSEs and the average computational cost of the three schemes mentioned above from 100 independent Monte Carlo runs. The improvement of *Scheme 3* in the position estimation was 4.55% w.r.t. *Scheme 2* and 8.11% w.r.t. *Scheme 3*. The velocity RMSEs are not given here due to the fact that the iteration algorithm mainly focused on addressing the linearization error caused by the linear approximation of the measurement function, and it can be seen from Equations (12) and (13) that triaxial velocities make no contribution to the measurement function in this case.

The computational cost of *Scheme 3* was 3.64% more w.r.t. *Scheme 1* and 18.96% less w.r.t. *Scheme 2*. The performance of the three schemes is summarized in Table 3, indicating that *Scheme 3* results in a good trade-off between position estimation accuracy and computational efficiency.

**Figure 11:**
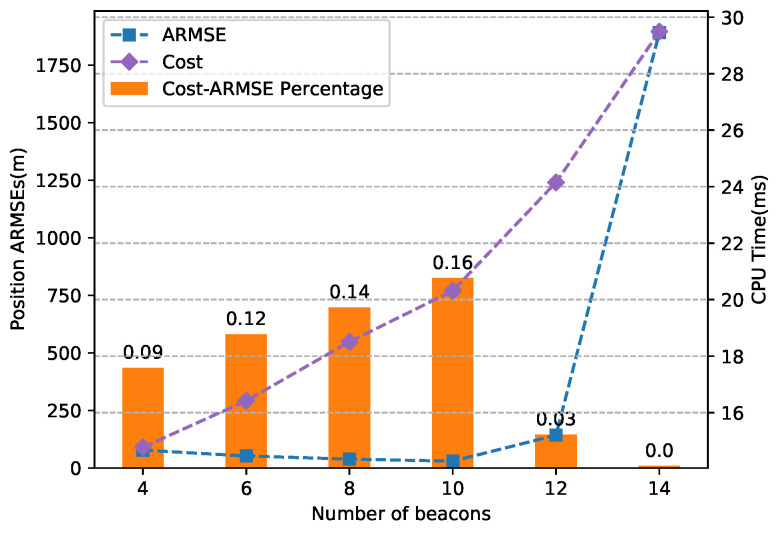
Comparison of the position estimation accuracy from different numbers of beacons.

The influence of the beacon number *n* is given in Figure 11. From the results, the position ARMSE continued to decrease when *n* was lower than 10 and increased to a large error in the order of kilometers, while the computational cost continued to increase with *n*. We defined an indicator call cost–ARMSE percentage, which was equal to 100/(Cost∗ARMSE) to further illustrate the results and indicated that n=10 was the most suitable configuration in our case.

**Figure 12:**
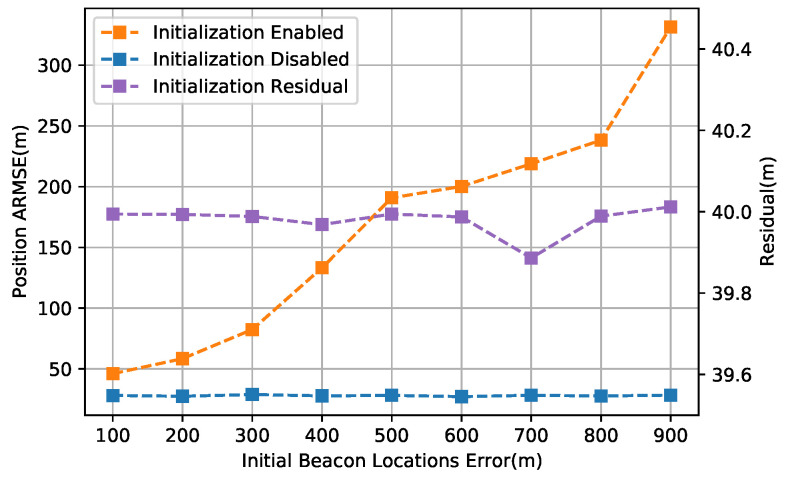
Comparison of the position estimation accuracy from different numbers of beacons.

Figure 12 gives the comparison of the proposed scheme under different initial location errors. We can infer from the results that the batch-least-square trilateration initialization algorithm achieved a consistent residual (around 40 m) regardless of the beacon initial location error in our application, which also led to a good robustness of the proposed localization scheme to the beacon initial noise. Extensive results with the initialization procedure disabled are further illustrated, which further prove the conclusion.

**Figure 13:**
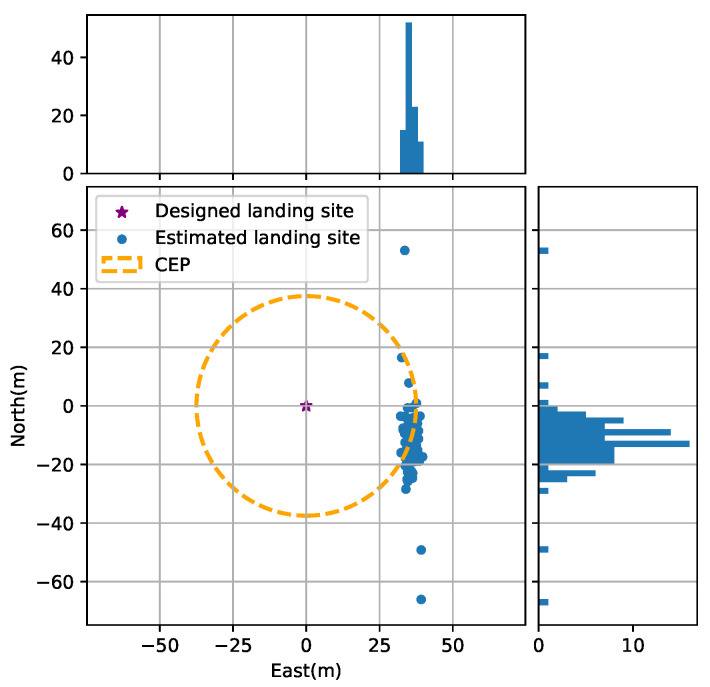
The circular error probable (CEP) of the proposed method from 100 independent Monte Carlo runs.

Finally, another 100 independent Monte Carlo runs were performed to evaluate the final drops of the proposed method, and the results are plotted in Figure 13, showing a circular error probable (CEP) of 37.52 m and a mean value of $\left[35.66\;m,-12.17\;m\right]$. Another interesting point is that the distribution of the final drops in the north (11.60 m) was much larger than that in the east (1.58 m). One possible reason for this phenomenon may lie in the geometrical configuration of all ground beacons, which has a significant effect on the observability of the whole system.

## 5. Conclusions

In this article, a distributed localization scheme was proposed to handle the situation of having no accurate prior knowledge regarding the beacon configuration in lunar pinpoint landing. This scheme integrated onboard IMU/altimeter measurements together with lander-beacon range measurements within a SEIF-based framework. The prediction and update stages of SEIF were replaced by a hybrid-form propagation and a damping iteration algorithm, respectively, which led to the so-called AISEHF. The simulation results indicated that the proposed scheme could satisfy the navigation demands of lunar pinpoint landing and showed a robustness to uncertain initial beacon locations. The Monte Carlo simulation indicated that our scheme makes a good trade-off between estimation accuracy and computational efficiency compared with the existing algorithms.

Although the research in this paper has proved to be significant, there are still some questions that require further investigation, such as the augmentation of the current range-only measurements with the range-rate and the optimization of the geometrical pattern of all ground beacons. Several realistic problems, such as transmission loss and glint noise, should be taken into consideration and verified by experiments. Our work will be expanded to include these issues in the future.

## Figures and Tables

**Table 1:** The true locations of all ground beacons.

Beacon ID	Locations in L (m)	Beacon ID	Locations in L (m)
1	$\left[-10,467.97,-1353.06,0\right]$	2	[−7647.32,1719.73,0]
3	$\left[-7245.89,-1587.17,0\right]$	4	$\left[-5465.18,2107.20,0\right]$
5	$\left[-5149.39,-3005.92,0\right]$	6	$\left[-2578.25,2027.77,0\right]$
7	$\left[-2145.57,-876.16,0\right]$	8	$\left[-421.43,2305.71,0\right]$
9	$\left[676.64,-2427.07,0\right]$	10	$\left[1649.64,1817.82,0\right]$

**Table 2:** Sensor parameters (1σ).

Parameter	Value	Parameter	Value
Accelerometer bias level	0.29 mg	Gyro bias level	$4.86\times 10^{-4}\;\mathrm{rad}/s$
Accelerometer random walk	$8.97\times 10^{-2}\;mg/\sqrt{Hz}$	Gyro random walk	$2.22\times 10^{-5}\;rad/\left(s\cdot \sqrt{Hz}\right)$
Accelerometer white noise	$2.14\times 10^{-3}\;mg/\left(s\cdot \sqrt{Hz}\right)$	Gyro white noise	$4.98\times 10^{-6}\;rad/\left(s^{2}\cdot \sqrt{Hz}\right)$
Beacon range noise	10 m	Altimeter range noise	0.5 m

**Table 3:** The performance of different algorithms.

Algorithm	Position Averaged RMSE (ARMSE) (m)	Velocity ARMSE (m/s)	CPU Times (ms)
Scheme 1	29.71	2.69	20.26
Scheme 2	28.60	2.75	26.06
Scheme 3	27.30	2.68	20.93

## Appendix A. Adaptive Iterated Update Algorithm

The detailed matrix form of $J_{i}$ is given as

(A1) $$\begin{array}{c} J_{i}={\left.\frac{\partial f(\xi_{t+1})}{\partial \xi_{t+1}}\right|}_{\xi_{t+1}^{i}}=-C{\left[H_{i}^{T},I\right]}^{T} \end{array}.$$

Substituting Equation (A1) into Equation (30) leads to

(A2) $$\begin{array}{cc} \xi_{t+1}^{i+1} & =\xi_{t+1}^{i}+{\left(H_{i}^{T}R^{-1}H_{i}+{\hat{\Lambda }}_{t+1}+\lambda I\right)}^{-1}\left[H_{i}^{T},I\right]\left[\begin{array}{cc} R^{-1} & 0\\ 0 & {\hat{\Sigma }}_{t+1}^{-1} \end{array}\right]\left[\begin{array}{c} z_{t+1}-h\left(\xi_{t+1}^{i}\right)\\ {\hat{\mu }}_{t+1}-\xi_{t+1}^{i} \end{array}\right]\\ \; & ={\left(H_{i}^{T}R^{-1}H_{i}+S\right)}^{-1}\left[H_{i}^{T}R^{-1}\left(z_{t+1}-h\left(\xi_{t+1}^{i}\right)+H_{i}\xi_{t+1}^{i}\right)+{\hat{\Sigma }}_{t+1}^{-1}{\hat{\mu }}_{t+1}\right] \end{array}$$

where $S={\hat{\Lambda }}_{t+1}+\lambda I$. Expanding the above equation with the matrix inversion lemmas given in [33], we can achieve

(A3) $$\begin{array}{cc} K^{i} & ={\left(H_{i}^{T}R^{-1}H_{i}+S\right)}^{-1}H_{i}^{T}R^{-1}\\ \xi_{t+1}^{i+1} & =K\left(z_{t+1}-h\left(\xi_{t+1}^{i}\right)+H_{i}\xi_{t+1}^{i}\right)+{\left(H_{i}^{T}R^{-1}H_{i}+S\right)}^{-1}{\hat{\Sigma }}_{t+1}^{-1}{\hat{\mu }}_{t+1}\\ \; & =\Gamma_{t+1}{\hat{\mu }}_{t+1}+K^{i}\left[z_{t+1}-h\left(\xi_{t+1}^{i}\right)-H\left(\Gamma_{t+1}{\hat{\mu }}_{t+1}-\xi_{t+1}^{i}\right)\right]\\ \Gamma_{t+1} & =\frac{{\hat{\Lambda }}_{t+1}}{\lambda }\left[I-{\left(I+\lambda {\hat{\Lambda }}_{t+1}\right)}^{-1}\frac{{\hat{\Lambda }}_{t+1}}{\lambda }\right]\\ \Lambda_{t+1}^{i} & =H_{i}^{T}R^{-1}H_{i}+{\hat{\Lambda }}_{t+1}. \end{array}$$
